# Multifunctional Vitamin B_1_-Derived Fluorescent Copper Nanoclusters for Efficient Maize Protoplast Transformation

**DOI:** 10.3390/antiox15081045

**Published:** 2026-08-21

**Authors:** Nikolett László, Milán Szabó, Györgyi Ferenc, Ditta Ungor

**Affiliations:** 1 Institute of Plant Biology, HUN-REN Biological Research Center, Temesvári Krt. 62, H-6726 Szeged, Hungary; laszlo.nikolett@brc.hu (N.L.); szabo.milan@brc.hu (M.S.); ferenc.gyorgyi@brc.hu (G.F.); 2 Doctoral School of Biology, University of Szeged, Dugonics Sqr. 13, H-6720 Szeged, Hungary; 3 Department of Physical Chemistry and Materials Science, University of Szeged, Rerrich B. Sqr. 1, H-6720 Szeged, Hungary

**Keywords:** copper nanocluster, nanozyme, biomimetic activity, antioxidant, peroxidase activity, cell treatment, ROS level, viability, transfection efficiency

## Abstract

Fluorescent copper nanoclusters are promising functional nanomaterials; however, controlling their redox behavior through ligand engineering remains challenging. Herein, a multifunctional Vitamin B_1_-derived fluorescent copper nanohybrid system was synthesized by a simple one-pot method, where Vitamin B_1_ served as both a reducing and stabilizing agent. Spectroscopic and structural characterization supported the formation of ligand-stabilized fluorescent ultrasmall copper species exhibiting blue emission at 465 nm with a quantum yield of 5.7%. The B_1_-Cu nanohybrid system displayed pronounced environment-dependent dual redox activity. ORAC analysis revealed a Trolox-equivalent antioxidant capacity of 229.2 ± 5.8 µM TE, compared with 28.1 ± 4.3 µM TE for pure Vitamin B_1_, while the ABTS assay yielded an IC_50_ value of 14.4 ± 0.4 µM, representing an approximately fivefold improvement over Vitamin B_1_ (73.7 ± 1.9 µM). In addition, the material exhibited pH-dependent peroxidase-like activity, demonstrating its nanozyme functionality. Biological validation in maize protoplasts showed concentration-dependent intracellular ROS regulation, a pronounced hormetic response, and up to 83% higher GFP-mediated transformation efficiency than the untreated control. These findings demonstrate that ligand-directed modulation of fluorescent copper nanoclusters provides an effective strategy for engineering multifunctional redox-active nanohybrid systems for plant biotechnology and other redox-regulated biointerface applications.

## 1. Introduction

Nowadays, the development of new nanomaterials with multifunctional properties is one of the most important aims in chemical sciences. These efforts should be focused on cost-effective and easy-to-implement ways to reach more and more eco-friendly options. One viable route is the synthesis of metal nanoclusters (NCs), which are generally defined as ultrasmall, sub-nanometer to low-nanometer scale metal-based clusters consisting of a small number of atoms, exhibiting molecule-like electronic structure and size-dependent fluorescence, often stabilized by organic ligands and showing synthesis-condition-tunable properties [1,2]. Besides the popular optical sensor applications [3,4], the biomimetic enzyme-like activity is one of the great behaviors of these small metals. In contrast to natural enzymes, they have several advantages such as high stability under wide pH and salt concentrations, reproducibility, scalability, and low cost. These properties can be utilized in the area of pollutant degradation [5,6], cancer treatment [7,8], antibacterial therapy [9] or in biosensing applications [10,11]. As can be seen in the past few years, the popularity of the copper-based nanozyme topic is also constantly growing [12]. Numerous natural oxidoreductase enzymes (e.g., Cytochrome C oxidase, Catalase, Laccase) have copper-based active centers, while Cu is a redox-active element that can change the oxidation states between Cu^+^ and Cu^2+^ in several biological redox reactions. Moreover, in combination with the appropriate stabilizing molecule, the synergistic multifunctional enzyme-mimic activity can also be achieved, which can facilitate the real-world usability [13,14,15,16]. Previously, several articles can be found about the CuS- and CuO-containing NCs [17,18] because they have great stability and low toxicity in living systems, but nowadays, the pure copper content is also a focus of interest. In most cases, peroxidase (POD) mimicking nanozymes can be found with higher and more specific sensitivities. For example, in the work of Hu et al., the bovine serum albumin-stabilized Cu NCs were presented, which have comparable catalytic activity to the natural horseradish POD enzyme [19]. This biomimetic property was taken advantage of in the development of a glucose sensor. Another glucose-sensing technology was established by Zhong and co-workers [20], where the NCs were stabilized by a thiolated β-cyclodextrin, which serves as a stabilizing molecule and effective modulator for the POD activity. Zhang et al. described that the albumin-biomineralized Cu NCs can serve as a great antioxidant platform in the case of oxidative stress [21].

Although the coordination chemistry of Vitamin B_1_ with transition-metal ions has been investigated for several decades [22,23,24], its potential as a multifunctional reducing and stabilizing ligand for fluorescent copper nanocluster-based systems has remained largely unexplored. Structurally, thiamine contains sulfur- and nitrogen-containing heterocyclic moieties that can coordinate Cu ions. Upon thermal oxidation, thiamine is converted into thiochrome and related oxidation products, providing a chemically plausible pathway for the reduction of Cu(II) species. Furthermore, the presence of multiple donor sites enables these oxidation products to participate in multidentate coordination, which may contribute to the stabilization of ultrasmall copper species during NCs formation [23]. The possible application as a stabilizing molecule of Au-based NCs has only been reported to a limited extent [25]. Our previous work [26] also investigated the Vitamin B_1_-Au NCs synthesis with this promising molecule, where we pointed out that the antioxidant behavior was increased by the presence of Au metal seeds.

Therefore, in this paper, we focus on the synthesis and characterization of copper-based thiamine-reduced NCs. In addition, the antioxidant and POD-like multifunctional activity was demonstrated by three different in vitro methods. We also investigated the effect of the Cu-based nanohybrid system on the ROS content, viability, and transfection efficiency of maize protoplasts. The results pointed out that the developed B_1_-Cu-nanohybrid system has a positive effect on the transfection efficiency, and since this is essential for the success of gene editing applications (e.g., CRISPR/Cas9), therefore they could be useful in gene editing-based applications in the future.

## 2. Materials and Methods

### 2.1. Chemicals

For the synthesis of B_1_-derived fluorescent Cu NCs, thiamine hydrochloride (Vitamin B_1_; C_12_H_17_ClN_4_OS·HCl; ≥99.9%; Sigma, Budapest, Hungary), copper(II) chloride dihydrate (CuCl_2_ × 2 H_2_O; 99.9% (metal basis); Sigma, Budapest, Hungary), and hydrochloric acid (HCl; 37%; Molar, Budapest, Hungary) were used. During the antioxidant measurements, 2,2′-azino-bis(3-ethylbenzothiazoline-6-sulfonic acid) diammonium salt (ABTS; C_18_H_24_N_6_O_6_S_4_; ≥98%; Alfa Aesar, Ward Hill, MA, USA), potassium persulfate (K_2_S_2_O_8_; 99.99%; Sigma, Budapest, Hungary), 2,2′-azobis(2-methylpropionamidine) dihydrochloride (AAPH; [=NC(CH_3_)_2_C(=NH)NH_2_]_2_ × 2 HCl; 97%; Sigma, Budapest, Hungary), 6-Hydroxy-2,5,7,8-tetramethylchromane-2-carboxylic acid (Trolox; C_14_H_18_O_4_; 97%; Sigma, Budapest, Hungary), and fluorescein sodium salt (C_20_H_10_Na_2_O_5_; analytical standard; VWR, Debrecen, Hungary) were selected. To prepare the suitable buffer solutions for the antioxidant measurements and protoplast treatment, the sodium dihydrogen phosphate 2-hydrate (NaH_2_PO_4_ × 2 H_2_O; 99%; Molar, Budapest, Hungary), disodium hydrogen phosphate dodecahydrate (Na_2_HPO_4_ × 12 H_2_O; ≥99.99%; Molar, Budapest, Hungary), sodium chloride (NaCl; 99.98%; Molar, Budapest, Hungary), acetic acid (CH_3_CO_2_H; 99%; Sigma-Merck KGaA, Darmstadt, Germany), sodium acetate trihydrate (CH_3_COONa × 3 H_2_O; 99.0%; Sigma-Merck KGaA, Darmstadt, Germany), citric acid monohydrate (HOC(COOH)(CH_2_COOH)_2_ × H_2_O; 99.6%; Molar, Budapest, Hungary), trisodium citrate dihydrate (C_6_H_5_Na_3_O_7_ × 3 H_2_O; 99.5%; Molar, Budapest, Hungary), dimethyl sulfoxide (DMSO; (CH_3_)_2_SO; ≥99.9%; Sigma-Aldrich, Darmstadt, Germany), 2′,7′-dichlorodihydrofluorescein diacetate (H2DCFDA; ≥97%; Sigma-Aldrich, Darmstadt, Germany), fluorescein diacetate (FDA; Sigma-Aldrich, Darmstadt, Germany), propidium iodide (PI; ≥94.0; Sigma-Aldrich, Darmstadt, Germany), polyethylenimine, branched (Sigma-Aldrich, Darmstadt, Germany), potassium dihydrogen phosphate (KH_2_PO_4_, Duchefa, Haarlem, The Netherlands), calcium chloride dihydrate (CaCl_2_·2H_2_O; Duchefa, Haarlem, The Netherlands), 2-(*N*-morpholino)ethanesulfonic acid (MES; Sigma-Aldrich, Darmstadt, Germany), magnesium sulfate heptahydrate (MgSO_4_ × 7H_2_O; Molar Chemicals, Halásztelek, Hungary), D(-)-mannitol (C_6_H_14_O_6_; Molar Chemicals, Halásztelek, Hungary) were applied. The reagents were used without further purification, and the stock solutions were prepared freshly with Milli-Q ultrapure water (Merck Millipore, Darmstadt, Germany; conductivity: 18.2 MΩ·cm). For the purification of the prepared NCs, a Pur-A-Lyzer^TM^ dialysis kit (Sigma, Budapest, Hungary) with a 1 kDa cut-off value was chosen.

### 2.2. Instruments

The optical characterization of the fluorescent nanoproducts was investigated by an ABL&E JASCO FP-8500 spectrofluorometer (ABL&E-JASCO Hungary Ltd., Budapest, Hungary) in a standard 1 cm quartz cuvette. The fluorescence spectra were registered using 2.5–2.5 nm bandwidths, 1 nm resolution, and 200 nm/min scan speed. During the further fluorescence investigations, the excitation and emission maxima were identified at 390 and 465 nm, respectively. The absolute internal quantum yield was determined using the same instrument equipped with an ABL&E JASCO ILF-835 (ABL&E-JASCO Hungary Ltd., Budapest, Hungary) integrating sphere and an ABL&E JASCO ESC-842 (ABL&E-JASCO Hungary Ltd., Budapest, Hungary) calibrated WI light source. The evaluation of the spectra was done by the SpectraManager 2.0 software provided by the manufacturer. The fluorescence lifetime of the NCs was determined by time-correlated single-photon counting (TCSPC) measurements on a Horiba DeltaFlex (Horiba Jobin Yvon Ltd., Palaiseau, France) apparatus using a 1 cm optical length. A DeltaDiode pulsed laser (λ_laser_ = 371 nm; Horiba Jobin Yvon Ltd., Palaiseau, France) was attached to the instrument, which served as an excitation light source. The emitted light was detected at 465 nm with a 6 nm slit. The number of counts on the peak channel was 10,000. To determine the instrument response function (IRF), standard LUDOX^®^ SiO_2_ colloids were applied, which were provided by the instrument manufacturer. The lifetime components were calculated by exponential fitting with χ^2^ goodness values of the registered decay curves in the EZTime program of Horiba Ltd. The fluorescence of the H2DCFDA dye in the biological samples was measured with a plate reader (Hidex Chameleon, v5.01; Hidex Oy, Turku, Finnland), in fluorescence mode. Fluorescence excitation/emission wavelengths were set to 485 nm/535 nm, respectively, using filters. The optical density was measured at 750 nm in absorbance mode, on the same samples. The instrument operation and data recording were performed using the MikroWin 2010 software (v5.12). The oxidation state of metal content, as well as the binding energies of the other elements in the prepared fluorescent nanoproducts, were analyzed by X-ray photoelectron spectroscopy techniques. The spectra were collected by a SPECS (SPECS GmbH, Berlin, Germany) device equipped with a PHOIBOS 150MCD9 hemispherical analyzer (SPECS Surface Nano Analysis GmbH, Berlin, Germany). The sample was deposited on a titanium foil having a thickness of 0.5 mm (Sigma-Aldrich, Hamburg, Germany) by multistep cyclic freeze-drying. The Al Kα X-ray source was operated at 200 W power, and the carbon 1s peak (284.80 eV) was used for charge referencing. Spectra were evaluated by CasaXPS software (version 2.3.24, Casa Software Ltd., Teignmouth, UK). In addition, to examine the primary coordination between the Vitamin B_1_ and Cu cores, Fourier-transformed Infrared spectroscopy (FT-IR) was used. The spectra were recorded with a JASCO FT/IR-4700 instrument equipped with an ATR PRO ONE Single-reflection accessory (ABL&E-JASCO Ltd., Budapest, Hungary). The spectra were collected in the range of 1000 to 4000 cm^−1^ by 256 interferograms with 1 cm^−1^ resolution on lyophilized powder samples. For the references, the powder of thiamine solution was prepared at the same concentrations and similar pH to the cluster dispersions, then the powder was produced by lyophilization. Before the in vitro antioxidant and enzymatic assays, the stability of the NCs was investigated by dynamic light scattering on a Malvern Zetasizer Nano ZS ZEN 4003 apparatus (Malvern Panalytical Ltd., Worcestershire, UK) at room temperature. The light source was a He-Ne laser (λ_laser_ = 633 nm), and the hydrodynamic diameters with the polydispersity index (PDI) were calculated by the Smoluchowski model. The transmission electron microscopy (TEM) images were captured on a FEI Tecnai G2 instrument (Thermo Fisher Scientific, Hillsboro, OR, USA) at 200 kV accelerating voltage. The samples were dried directly onto the carbon film-coated copper grids. The images were analyzed by ImageJ (version 1.54g; U. S. National Institute of Health, Bethesda, MD, USA), open-source software. The enzymatic reactions were investigated by UV-Vis and fluorescence spectroscopy. The fluorescence spectra were recorded on the above-mentioned spectrofluorometer. While the UV-Vis spectra were recorded on an ABL&E JASCO V-770 spectrophotometer (ABL&E-JASCO Hungary Ltd., Budapest, Hungary) between 300 and 600 nm with 1 nm resolution and 200 nm/min scan speed during the static measurements, the 470 nm wavelength was chosen for the time-dependent measurements at 20 ± 0.1 °C. For the measurements, standard 1 cm quartz cuvettes were used. The spectra were analyzed in the ABL&E JASCO SpectraManager 2.5 software (ABL&E-JASCO Hungary Ltd., Budapest, Hungary). Visitron spinning disk microscope was used for viability test imaging and counting the green fluorescent protein (GFP)-positive cells. A 488 nm excitation laser was used for FDA and GFP; a 543 nm laser was used for PI. FDA and GFP emission were detected between 505 and 530 nm, and PI emission was detected between 570 and 670 nm. Fluorescence intensity was determined using Infra-View software (infraView GmbH, Mainz, Germany).

### 2.3. Synthesis of the Vitamin B_1_-Derived Cu Nanohybrid System

During the preparation of the fluorescent copper-containing bioconjugates, the initial concentrations of Vitamin B_1_ and Cu in the reaction mixture were 31.25 and 1.25 mM, respectively, which means that the applied molar ratio was B_1_:Cu/25:1. For the first step, the suitable amounts of the powdered B_1_ and CuCl_2_ were dissolved in ultrapure water. In addition, the pH of the B_1_ solution was adjusted to pH 3.0 with a 10 mM HCl solution. After mixing and further 5 min of magnetic stirring of the solutions, the sample was thermostated at 80 °C for 1 day. During the synthesis, the original bluish color changed to yellow with some dark brown aggregates, which were removed by centrifugation at 13,000 rpm for 15 min and dialysis for 2 h in a Pur-A-Lyzer^TM^ Mega 1000 dialysis kit (MWCO = 1 kDa; Sigma-Aldrich, Budapest, Hungary).

After the purification, the total copper amount of the sample was 1.12 ± 0.03 mM by inductively coupled plasma mass spectrometry (ICP-MS measurements were performed on an Agilent 7700x type quadrupole instrument at the University of Szeged, Szeged, Hungary). The final Vitamin B_1_ concentration was 20.2 ± 0.2 mM by UV-Vis spectrophotometry, which results in a ca. B_1_:Cu/18:1 molar ratio in the final samples.

### 2.4. Study of the Biomimetic Antioxidant and Peroxidase Activity

The biomimetic antioxidant activity was analyzed using the Oxygen Radical Antioxidant Capacity (ORAC) assay, which serves to detect the presence of the chain-breaking antioxidant mechanism. For this purpose, the degradation of the generally used fluorescein dye was monitored by spectrofluorometry. The emission spectra were recorded between 500 and 600 nm, while 515 nm and 485 nm were chosen for the emission maxima and excitation wavelength, respectively. For the measurements, a final 50 µM concentration of B_1_, B_1_-Cu bioconjugate or Trolox was used in individual samples; 78 μL of fluorescein solution (c = 1 μM) was also pipetted into phosphate-buffered saline (PBS, c_NaCl_ = 0.15 M, pH 7.4). Before the start of the reaction, the samples were thermostated at 37 °C for 15 min in the dark. After the incubation, the application of 100 μL of 200 mM AAPH solution started the reaction. To reach a suitable fluorescence signal, the final volume of the samples was adjusted to 2 mL. Before the calculation, the antioxidant capacity was characterized by the *ORAC value* [27] based on Equation (1), the hydrophilic Vitamin E derivative Trolox molecule was selected for reference:

(1) $$ORAC\;value=\;\frac{c_{Trolox}\;\times \;{Net}_{AUC\;of\;sample}\;\times \;k}{{Net}_{AUC\;of\;Trolox}}$$

where *c_Trolox_* is 50 μM, the *Net_AUC_* of the sample and *Net_AUC_* of Trolox values are the net areas under kinetic curves in the case of 50 μM B_1_ content in the sample and 50 μM in the Trolox solution. Each *Net_AUC_* value was calculated after blank correction by integrating the time-dependent decay curves. The *k* is the dilution factor of the sample, which was 15-fold in our case. The final values were defined as µM TE (Trolox equivalent).

Secondly, the standard 2,2′-azino-bis(3-ethylbenzothiazoline-6-sulfonic acid) (ABTS) method was selected, by which the total antioxidant capacity can be determined. For the measurements, a 10 µL sample and 200 µL ABTS working solution were mixed. After 5 min dark incubation at 25 °C, the liquids were diluted to 3 mL, and the UV-Vis spectra were recorded. The absorbance was read at 734 nm in each case, and the antioxidant capacity was determined by the IC_50_ value, where the *Scavenging*% reached 50% based on the following formula (Equation (2)) and linear interpolation [28]:

(2) $$Scavenging\;\%=\left(\frac{A_{c}-A_{s}}{A_{c}}\right)\times 100$$

where *A_c_* and *A_s_* are the absorbance at 734 nm of the control and sample after 5 min, respectively. The samples were investigated in the 1–100 μM concentration range.

For the identification of the peroxidase (POD) enzyme activity, two measurement protocols were selected [29,30], where the reduction of H_2_O_2_ to water is the model reaction in the presence of TMB dye. Firstly, so-called stationary measurements were applied, where the oxidation of TMB was stopped. The final reaction mixture was prepared by blending 40 µL TMB solution (dissolved in DMSO at a 10 mg/mL concentration) and a suitable amount of the tested sample in PBS at pH 7.4, citrate buffer at pH 3.6, and sodium bicarbonate buffer at pH 10.1. To start the reaction, a freshly prepared 155 µL H_2_O_2_ solution was added to the mixture to adjust its 5 mM concentration; therefore, the final volume of the reaction mixture reached 3 mL. After 10 min at room temperature, the reaction was stopped by adding 200 µL of 0.1 M H_2_SO_4_, and the UV-Vis spectra were recorded between 300 and 600 nm, where the absorbance was read at 450 nm. During the kinetic measurements, the reaction could not be stopped; therefore, the appearance of blue colored oxidized TMB was monitored at 647 nm for 30 min at 20 °C. In this case, the examined amount of the samples was fixed, and the concentration of H_2_O_2_ was varied between 5 and 25 mM. To analyze the POD activity, the kinetic parameters (*K_M_*, *v_max_* and *k_cat_*) were obtained by nonlinear regression fitting of the Michaelis–Menten equation using OriginPro 2018 (OriginLab Corporation, Northampton, MA, USA).

### 2.5. Measurements of the Effect of B_1_-Cu Bioconjugate on the ROS Content, Viability, and Transfection Efficiency of Maize Protoplasts

#### 2.5.1. Isolation of Maize Protoplasts and Treatment of Protoplasts with Vitamin B_1_ or Cu-Containing System

*Zea mays* L. (SZ22 and H1233) cell suspensions provided by Sándor Mórocz (Cereal Research Institute, Szeged, Hungary) were cultured in N6M medium in the presence of 0.5 mg/L 2,4-dichlorophenoxyacetic acid (2,4-D). Isolation of protoplasts was performed as described by [31] Mórocz et al. The cell wall was digested overnight with a mixture of cellulase RS and Pectolyase Y23; after filtration with a 60 µm sieve and centrifugation at a standard 1000 rpm, the pellet was washed 3×. At the end of the isolation, protoplasts were suspended in “R-medium” (0.75M mannitol, 1 mM CaCl_2_, 4 mM MgSO_4_, 620 mM KH_2_PO_4_, and 3.07 mM MES at pH 5.7). Vitamin B_1_ or B_1_-Cu nanohybrid system was tested in the concentration range from 10 µM to 360 µM by diluting the stock solutions with “R-medium” in a final volume of 225 µL. These were added to a 25 µL maize protoplast suspension.

#### 2.5.2. Measuring the ROS Content of the Protoplast Suspensions [32]

50 µL of the protoplast suspensions from the previous step were transferred to a 96-well plate. 4 µL stock solution of H2DCFDA (0.1 mg/mL in DMSO) was added to each sample, and their fluorescence was measured with a plate reader (Hidex Chameleon, v5.01; fluorescence excitation/emission wavelengths set to 485 nm/535 nm, respectively) after 1.5 h of incubation.

#### 2.5.3. Determination of the Viability of the Protoplasts [33]

The FDA/PI viability assay was performed after 1.5 h of incubation of protoplasts with vitamin B_1_ or Cu NCs. Before use, PI (0.013 mg/mL in DW) and FDA stock solutions (5 mg/mL in DMSO) were diluted 10× with R-medium, immediately added to the protoplast samples, carefully mixed, and examined under the Visitron spinning disk microscope (Visitron Systems GmbH, Puchheim, Germany).

#### 2.5.4. Transformation of Maize Protoplasts with pGFP Plasmid via Cationic Polymer in the Presence of Different Concentrations of Vitamin B_1_ and B_1_-Cu Nanohybrid System

According to the protocol described by Nagy B. et al. [34], the pGFP plasmid was delivered into protoplasts via a cationic polymer in the presence of Vitamin B_1_ or B_1_-Cu nanohybrid system at different concentrations. Transformation in brief: The pGFP plasmid and cationic polymer were incubated for 30 min in R medium to form a polyplex, then it was added to the protoplast suspension containing Vitamin B_1_ or B_1_-Cu bioconjugate and incubated for another 30 min at room temperature. At the end, the supernatant was removed, and fresh ppN6M was added to the wells. This step was repeated twice. After 4 and 15 days, three samples from each treatment were examined under a Visitron spinning disk microscope to count GFP-positive maize cells.

### 2.6. Statistical Analysis

Statistical analyses were performed using OriginPro 2018 (OriginLab Corporation, Northampton, MA, USA). Data are presented as mean ± standard deviation (SD) from at least three independent experiments. Pairwise comparisons between Vitamin B_1_ and the B_1_-Cu nanohybrid system at corresponding concentrations (ORAC, ABTS, POD activity, ROS content, and viability assays) were performed using Welch’s two-tailed *t*-test. GFP transformation efficiency data were analyzed by two-way analysis of variance (two-way ANOVA included treatment and concentration). Differences were considered statistically significant at *p* < 0.05.

## 3. Results and Discussion

### 3.1. Optical and Structural Properties of the Fluorescent B_1_-Cu Bioconjugate

In the first step of developing new fluorescent materials, the synthesis protocol should be worked out because the key parameters have a great influence on the formed nanostructure. In this case, the initial molar ratios of the copper(II) ion and Vitamin B_1_, the applied metal concentration, the starting pH of the reaction mixture, the temperature, as well as the synthesis time were investigated, and the ideal parameters resulted in the highest fluorescence intensity. For this purpose, the synthesis was completed by using a B_1_:Cu/25:1 molar ratio with 1.25 mM copper(II) concentration; the initial pH of the thiamin solution was pH = 3.0, and the temperature was adjusted to 80 °C for 24 h. After purification by dialysis, the blue fluorescence of the purified B_1_-Cu system was analyzed by UV-Vis spectrophotometry, spectrofluorimetry, and TCSPC methods. In the UV-Vis spectrum (Figure S1a), the presence of Cu nanoparticles is not identifiable based on the lack of a characteristic plasmon band between 560 and 590 nm. Therefore, the optical response is consistent with ultrasmall Cu species rather than plasmonic Cu nanoparticles (NPs).

**Figure 1:**
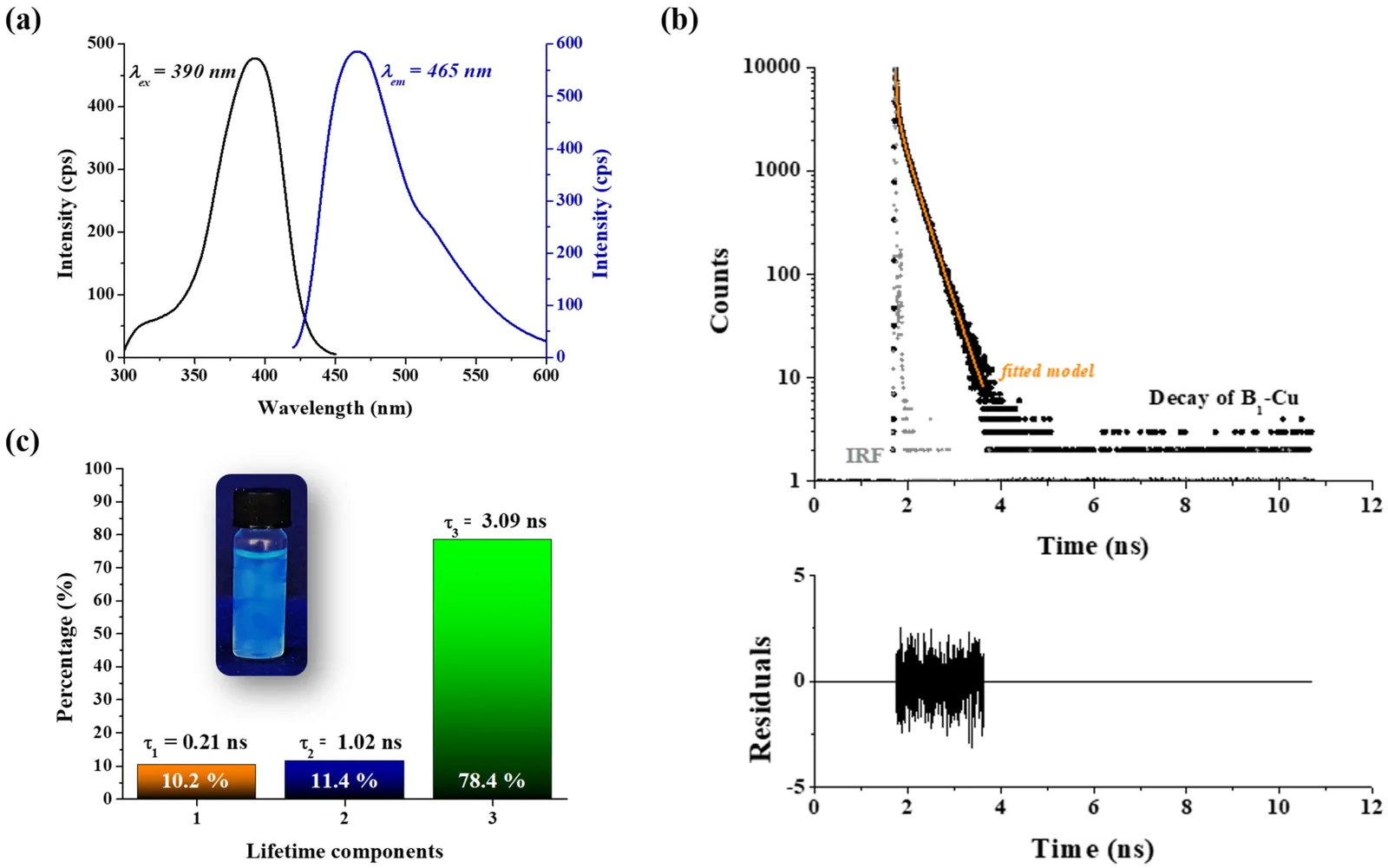
(**a**) Excitation and emission spectra of the B_1_-Cu nanohybrid system. (**b**) The fluorescence decay curve of the B_1_-Cu nanohybrid system (**black dots**) with the instrument response function (**gray dots**) and the fitted three-exponential-based model (**orange line**). (**c**) Relative contribution of the calculated lifetime components with the photo of the sample under a UV lamp (λ_lamp_ = 365 nm).

As can be seen in Figure 1a, the typical excitation and emission spectra show maximal values at 390 and 465 nm, respectively. The large Stokes shift (75 nm) is in good agreement with emissive Cu nanocluster-type species. In addition, a broad emission with a strong asymmetric red shoulder can be observed, and the absolute quantum yield is 5.7 ± 0.2%. The combined presence of these features suggests the heterogeneity of the oxidation state of copper in the few-atom metal cores. The optical properties of ultrasmall metal NCs are often interpreted using the Jellium model, which relates the electronic structure of few-atom metallic species to their emission energy. Although this model cannot provide a direct structural assignment, it offers useful spectroscopic context for comparing the present system with previously reported fluorescent Cu NCs [35,36]. The measured emission energy (2.67 eV) and QY % fall within the range reported for several fluorescent few-atom Cu NC systems in the literature [36,37]. Taken together, the spectroscopic observations consistently support the formation of fluorescent ultrasmall Cu NCs, while the exact atomic structure cannot be resolved solely from optical measurements.

To further elucidate the emissive behavior, TCSPC measurements were also performed. The average fluorescence lifetime, as well as the main lifetime components with their contribution, were also calculated. A typical fluorescence decay curve can be seen in Figure 1b, while the contribution percentage of the lifetime components can be found in Figure 1c. For the prepared blue-emitting copper-based system, the average fluorescence lifetime is 1.16 ± 0.27 ns, which also supports the formation of a few-atomic-cluster-type complex structure [38]. For this average lifetime, three main lifetime components dominate, which are τ_1_ = 0.21 ns (10.2%), τ_2_ = 1.02 ns (11.4%), and τ_3_ = 3.09 ns (78.9%).

Considering the formation of cluster cores, it can be determined that while the Cu(II) ions are partially or completely reduced, the applied ligand is capable of so-called metal ion-induced oxidation, resulting in the formation of thiochrome molecules [39]. However, as discussed above, the contribution of free thiochrome to the overall fluorescence is considered limited under the applied conditions (synthesis at pH 3.0 and high Cu:B_1_ molar ratio), and therefore the dominant emissive pathway is suggested to originate from metal core–ligand associated states. However, distinguishing cluster-centered photoluminescence from potential emission from molecular thiochrome byproducts is a critical point. To separate these contributions, steady-state and time-correlated PL analyses were systematically performed in aqueous media. As shown in Figure S1b, pure molecular thiochrome exhibits a characteristically distinct optical fingerprint with excitation/emission maxima at 407 nm/495 + 525 nm. In contrast, the synthesized B_1_-Cu nanohybrid system exhibits a distinct optical fingerprint with excitation and emission maxima at 390 and 465 nm, respectively, both shifted relative to molecular thiochrome measured under identical pH conditions. The observed differences in both the excitation and emission profiles indicate that the optical response of the B_1_-Cu nanohybrid system differs from that of molecular thiochrome measured under identical conditions. These spectral differences are consistent with the formation of new emissive metal–ligand electronic states rather than fluorescence arising exclusively from free molecular thiochrome [40]. In addition, the average lifetime of free molecular thiochrome was 1.64 ± 0.03 ns. Based on this, the τ_2_ component may be associated with emissive ligand-centered states in the immediate coordination environment of copper, potentially involving oxidized thiamine-derived motifs [41]. The τ_1_ component reflects faster relaxation processes related to metal-centered or core-localized relaxation, while the most significant τ_3_ likely corresponds to the ligand-centered p band intermediate state [42]. In coordination chemistry, proximity to paramagnetic copper ions drastically accelerates non-radiative relaxation, shortening organic ligand lifetimes. The nearly twofold elongation of the dominant lifetime (3.09 ns) compared to the free fluorophore (1.64 ns) suggests that the dominant radiative recombination involves state-hybridized ligand-to-metal charge-transfer (LMCT) pathways within a ligand-stabilized Cu(I)-rich coordination environment rather than being explained solely by free molecular oxidation products [43]. Overall, the observed lifetime distribution supports the presence of a structurally dynamic, ligand-stabilized Cu NC system, where emissive properties arise from coupled metal–ligand electronic interactions rather than a single isolated molecular species. While the formation of highly stable few-atom copper cluster configurations may be considered, the present spectroscopic data do not allow direct atomic-level structural assignment.

**Figure 2:**
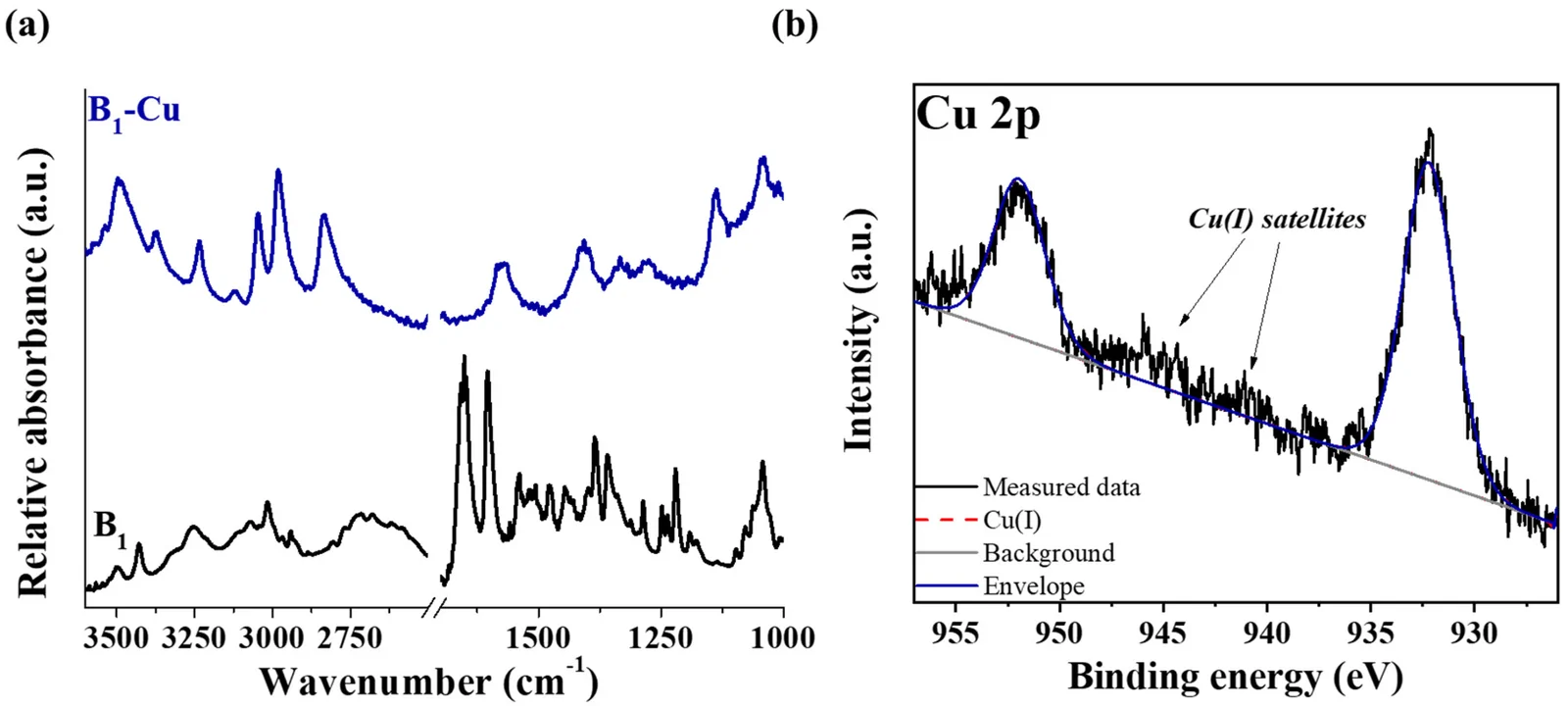
(**a**) The FT-IR spectra of the B_1_ (**black**) and B_1_-Cu nanohybrid system (**blue**). (**b**) High-resolution XPS spectrum of the Cu 2p region of the B_1_-Cu nanohybrid system.

For further characterization, the structural analysis was carried out by infrared spectroscopy and X-ray photoelectron spectroscopy. As can be seen in Figure 2a, the IR spectrum of Vitamin B_1_ is drastically changed due to the oxidation of the thiamin molecules and the formation of a copper-ligand coordination environment [44]. The changes in the lower wavenumbers also support the possible coordination through Cu-S and Cu-N electronic coupling.

For further investigations, the survey spectra of XPS measurements (Figure S2a) were carried out, indicating the presence of N, O, C, S, Cl, and Cu atoms in the B_1_-Cu nanohybrid system. In Figure 2b, the XPS spectrum from a high-resolution (HR) scan of copper content can be seen. Based on the fitted Cu 2p region, the absence of the Cu(II) shake-up satellites is consistent with the partial or complete reduction of the initially added Cu(II) ions. The 2p_3/2_ and 2p_1/2_ positions were 932.2 eV and 952.0 eV, respectively [45]. Therefore, the spectra indicate that Cu(I) is the predominant copper species in the investigated sample. The XPS results suggest the coexistence of copper atoms with slightly different electronic environments, consistent with a heterogeneous Cu(I)-rich coordination structure.

To identify the stabilizing bonds, one should also look at the XPS spectra of the other atoms. The N 1s spectrum (Figure S2b) consists of three major components. The peak at lower binding energy at 398.4 eV belongs to the imine nitrogen. The peak at 399.5 eV is assigned to the amine group, while the peak at 400.9 eV refers to the presence of quaternary amine nitrogen. The measured values are consistent with ligand-to-metal electronic interactions mediated through Cu-N coordination [46]. Both oxygen and carbon (Figure S2c,d) do not show any significant differences, but the spectrum of sulfur (Figure S2e) in the S 2p region shows two different components at 164.4 eV and 167.5 eV, where the lower energy value refers to coordinated aromatic ‘S’ atoms [47]. The 167.5 eV peak perhaps belongs to the partially oxidized thiochrome on the sample surface [48]. In addition, the Cl 2p region of chloride (Figure S2f) also appears as two different species at 196.7 eV and 199.0 eV, which refer to the weakly coordinated surface chloride and the strong Cu(I)-Cl bond [49]. Taken together, the FT-IR and XPS results support the formation of a Cu(I)-rich ligand-stabilized fluorescent copper nanocluster system. The spectroscopic data further suggest that oxidized Vitamin B_1_-derived species contribute to the coordination environment, while chloride ions participate in the stabilization of Cu(I) centers. Considering the combined spectroscopic observations, the obtained results are consistent with previously reported few-atom fluorescent Cu NCs. However, the present data do not allow an unambiguous atomic-level structural assignment; therefore, Scheme S1 should be regarded as a conceptual representation of the proposed coordination environment rather than an atomically resolved structural model.

To complement this conceptual model, the structural characteristics of the system were further investigated using TEM and DLS, which provide experimental insight into different hierarchical levels of the Cu–thiamine ensemble. It should be noted that the Cu–thiamine system represents a dynamic, hierarchical system rather than a single structurally rigid classical nanoparticle. To analyze the distinct length scales of the prepared Cu-based bioconjugate, different characterization techniques probe was used: TEM reflects dehydrated, aggregated domains, whereas DLS reports hydrodynamic diameters (d_H_) of solvated, polydisperse species. The emissive properties, however, originate from ultrasmall Cu emissive species that cannot be directly resolved by either TEM or DLS. The observed 1–2 nm particles in TEM (Figure S3a) images are interpreted as ligand-rich nanoscale domains rather than isolated crystalline nanoparticles. For the analysis of the stability of the prepared NCs, DLS and PL measurements were applied to record d_H_, the ζ-potential, and the fluorescence maximum values depending on the pH. The results can be seen in Figure S3b–d, as well as Table S1. The polydispersity index (PDI) values were consistently above 0.2 across the investigated pH range, indicating a polydisperse system. The larger loosely associated aggregates and the tendency of the changes depending on the pH were detectable. Based on our measurements, significant changes were observed in strongly acidic (pH < 2.0) media, where the samples lost their fluorescence, and several large (d_H_ ~ 200 nm) black aggregates were formed; meanwhile, the ζ-potential was close to 0 mV. In addition, an aggregation-induced emission (Figure S3c) was detected around pH 6–7, where the measured d_H_ reached ca. 50–70 nm, and the fluorescence also increased about twofold. At this pH, excess thiamine-derived ligands forming the outer coordination shell become less charged owing to pseudobase formation. Therefore, the observed aggregation-induced emission is consistent with restricted intramolecular motions within the ligand shell surrounding the emissive Cu-based species. The alkali conditions had no considerable effect on the stability. It should be emphasized that the hydrodynamic diameters obtained from DLS reflect solvated, dynamically assembling Cu–ligand aggregates rather than the size of the primary emissive Cu species. Therefore, the observed pH-dependent changes in d_H_ are consistent with changes in supramolecular organization of Cu-ligand system rather than changes in the intrinsic dimensions of the emissive species.

### 3.2. Determination of the Antioxidant Capacity and the POD-like Behavior

Although Vitamin B compounds are not generally considered strong classical antioxidants, several members of this family, including thiamine derivatives, have been reported to influence oxidative stress responses and contribute to cellular redox homeostasis. Therefore, the antioxidant properties of the B_1_-Cu nanohybrid system were further evaluated using two complementary assays. In our previously published work, the B_1_-Au NCs were also examined, where the same ORAC and ABTS techniques were used [26]. This work demonstrated that the presence of metal species significantly enhanced the antioxidant capacity compared with the pure thiamin molecule.

**Figure 3:**
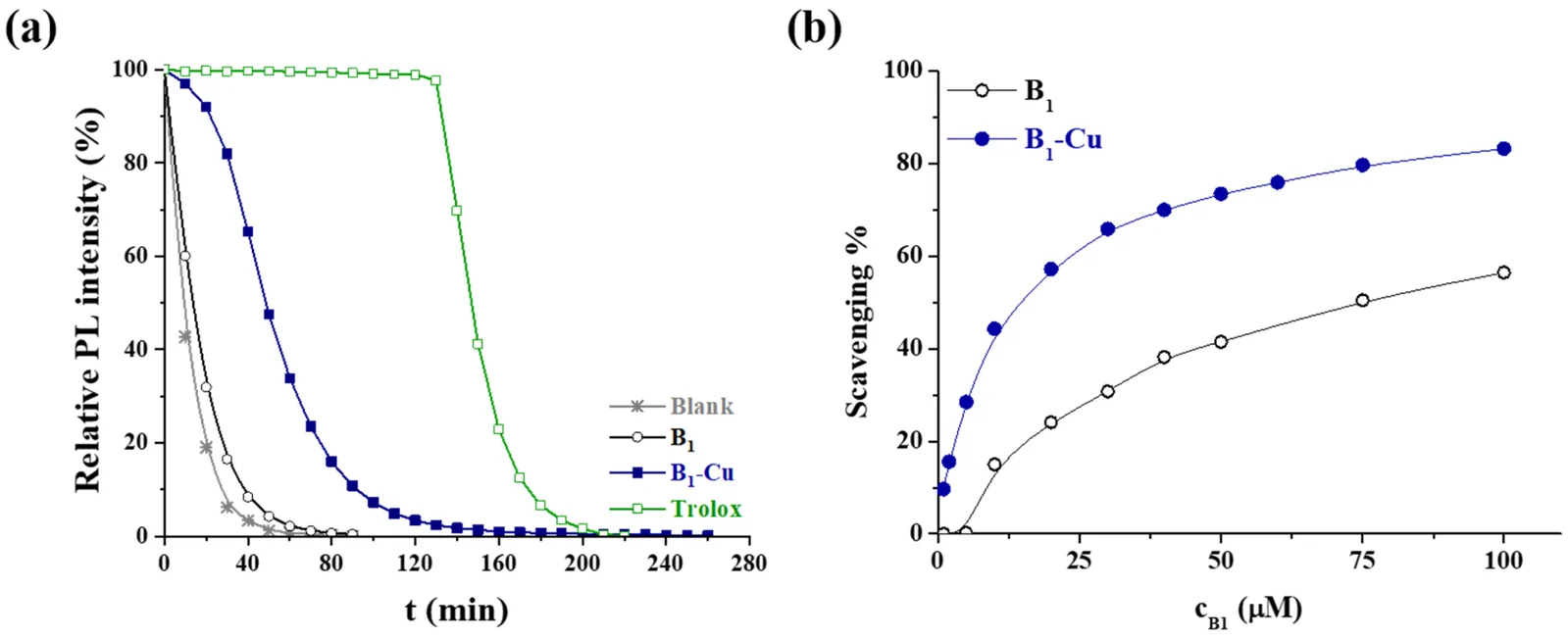
(**a**) The representative ORAC fluorescence decay curve obtained in the presence of Vitamin B_1_ (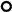), B_1_-Cu nanohybrid system (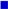) and Trolox (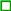) at 50 µM concentration with the blank (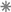). (**b**) ABTS radical scavenging activity of Vitamin B_1_ (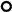) and B_1_-Cu nanohybrid system dispersion (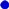) over the concentration range of 1–100 µM. [The applied concentrations refer to the Vitamin B_1_ content in both assays].

Firstly, the ORAC assay was used, where the scavenging of peroxyl radicals was studied. For this purpose, AAPH was used as a peroxyl radical generator, while the degradation of fluorescein was monitored by fluorimetry. As can be seen in Figure 3a, the prepared B_1_-Cu NCs show much better antioxidant activity than the pure B_1_ because the calculated relative *ORAC value*s are 229.2 ± 5.8 µM TE and 28.1 ± 4.3 µM TE, respectively, representing a statistically significant difference (Welch’s two-tailed *t*-test, *p* < 0.01, *n* = 3). Accordingly, the B_1_-Cu nanohybrid system exhibited a substantially higher Trolox equivalent antioxidant response under identical assay conditions. Thus, the B_1_-Cu system exhibited ca. 8-fold higher peroxyl radical-scavenging capacity in this in vitro study compared with the pure Vitamin B_1_. As a complementary antioxidant assay, the ABTS radical scavenging method was selected, where the decrease in absorbance of the pre-formed ABTS^•+^ radical solution was monitored spectrophotometrically at 734 nm as a function of antioxidant concentration. Based on the typical scavenging activity curve in Figure 3b, it can be determined that the IC_50_ value of the B_1_-Cu nanohybrid system, by linear interpolation, was 14.4 ± 0.4 µM, while the pure Vitamin B_1_ exhibited an IC_50_ value of 73.7 ± 1.9 µM, representing a statistically significant difference (Welch’s two-tailed *t*-test, *p* < 0.01, *n* = 3). The calculated IC_50_ values indicate that the prepared fluorescent B_1_-Cu nanohybrid system exhibited a significantly lower IC_50_ value compared to Vitamin B_1_, corresponding to an approximately fivefold higher ABTS radical-scavenging capacity. Overall, the results obtained from both antioxidant assays demonstrate a substantial enhancement in radical-scavenging activity after formation of the B_1_-Cu nanohybrid system, in agreement with the antioxidant properties reported for other fluorescent nanoclusters (Table 1).

**Figure 4:**
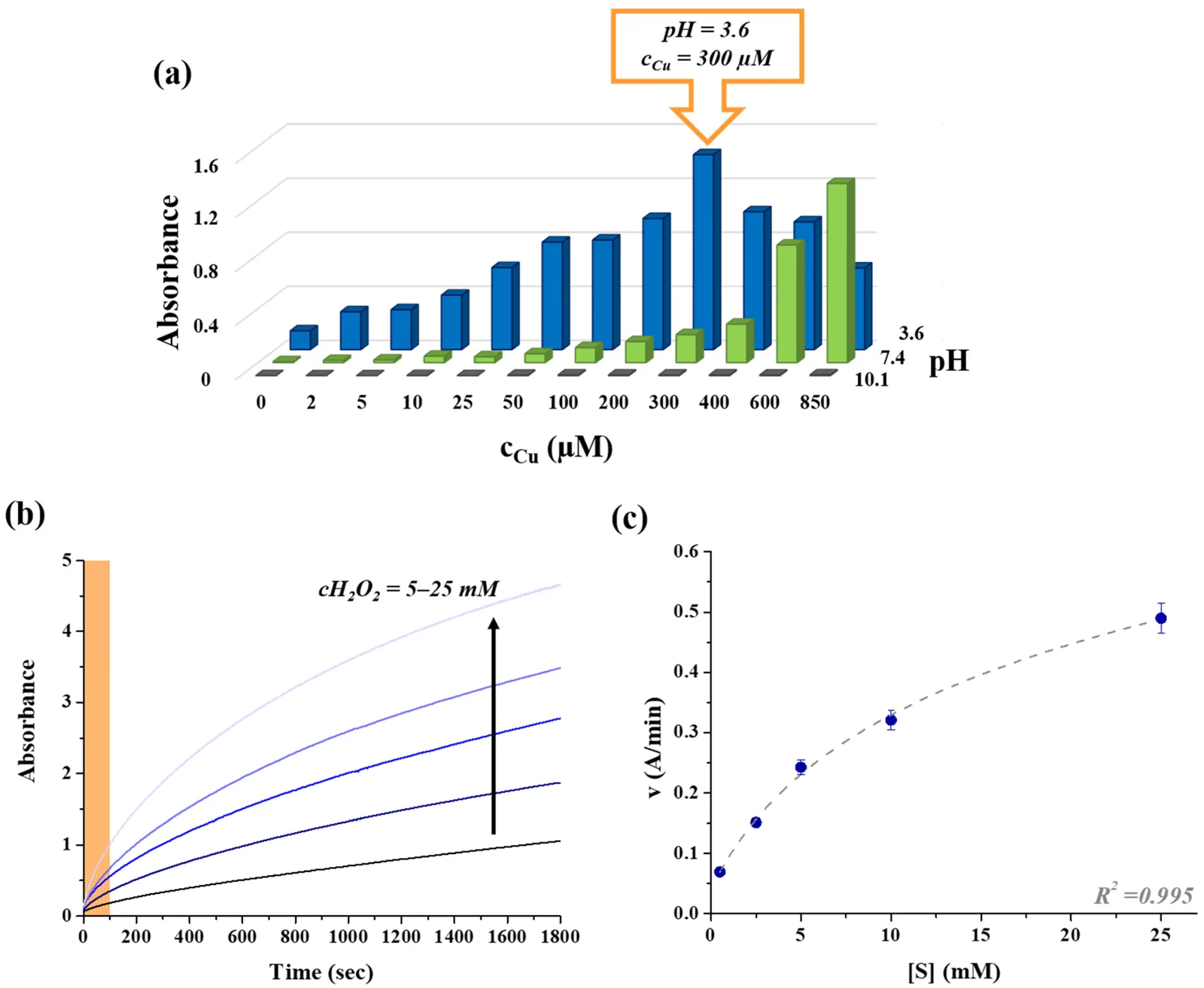
(**a**) Maximum absorbance at 450 nm as a function of different Cu concentrations at different pH values by stop-bath TMB assay. (**b**) The representative kinetic profile of the TMB oxidation catalyzed by the B_1_-Cu nanohybrid system (t = 20 °C; c_Cu_ = 300 µM; λ_abs_ = 647 nm; cH_2_O_2_ = 5–25 mM) with the linear fitting range (**orange section**). (**c**) Michaelis-Menten plot obtained from the kinetic measurements.

Given that Vitamin B_1_ is an efficient singlet oxygen scavenger and shows a notable activity against lipid peroxidation [56], the peroxidase-mimicking (POD) catalytic activity of the B_1_-Cu nanohybrid system was further investigated to evaluate its potential dual redox behavior. For this purpose, a simple, well-established TMB-based colorimetric assay was employed using two complementary experimental approaches. In this reaction, the oxidation of the TMB molecule was used as an indicator, while the H_2_O_2_ was reduced to water. Firstly, the peroxidase-like activity was evaluated using a fixed-time colorimetric assay, where the reaction was stopped by H_2_SO_4_ after 10 min at room temperature. Measurements were performed at pH 3.6 and pH 7.4 to compare acidic and physiological conditions. An additional measurement was carried out at pH 10.1 to evaluate the catalytic activity under alkaline conditions, where the B_1_-Cu nanohybrid system remains stable. The results of the measurements can be seen in Figure 4a, where the detected yellow absorbance is depicted against the applied Cu concentrations. The measurements indicate that it can be concluded that the prepared B_1_-Cu nanohybrid system exhibits pronounced pH-dependent POD-like activity, with the highest catalytic activity observed under acidic conditions. In contrast, the NCs show only moderate activity at physiological and alkaline conditions. The highest catalytic response was observed at pH 3.6 using a 300 µM Cu concentration. The observed pH dependence is consistent with the redox chemistry of Cu(I)/Cu(II) species involved in peroxidase-like catalysis. The Cu-based nanozyme activity is consistent with a pH-dependent Cu redox cycling process commonly described for Fenton-like catalytic mechanisms. Examination of the pure Vitamin B_1_ molecule revealed that thiamin did not show a significant effect under the same conditions (Figure S4). To further characterize the catalytic behavior, the steady-state enzyme kinetic experiments were performed under the selected catalytic optimum conditions.

During the kinetic measurements, the appearance of the blue oxidized TMB form was detected using individual samples with the presence of 0.5–25 mM of H_2_O_2_ concentrations. The analysis of the measurement data (Figure 4b) was carried out by fitting the linear range of the registered curves (*orange section*). After nonlinear regression fitting of the Michaelis–Menten model (Figure 4c), the apparent kinetic parameters were obtained as *K_M_* = 8.60 ± 0.43 mM, indicating a typical nanozyme-type low apparent substrate affinity due to heterogeneous surface binding. The *v_max_* is 2.75 ± 0.23 × 10^−4^ mM s^−1^. Based on the applied enzyme concentration, the apparent calculated *k_cat_* = (9.2 ± 0.78) ×10^−4^ s^−1^, which corresponds to an apparent catalytic turnover time of approximately 18 min per active site equivalent. This kinetic parameter clearly refers to a nanozyme characteristic surface-limited catalytic process, where the electron transfer is slow and limited [57,58,59]. It should be mentioned that the Michaelis-Menten formalism is used here as an empirical description of the observed saturation kinetics. The calculated parameters are apparent values, which refer to a finite number of catalytically active sites instead of exact proof of classical enzyme-substrate complex formation.

The combined antioxidant and POD-like activity measurements demonstrate that the B_1_-Cu nanohybrid system exhibits environment-dependent dual redox behavior. This property is consistent with the Cu(I)-rich nature of the system and dynamic surface redox processes. Under ROS-rich conditions, the Cu(I)-enriched surface sites are expected to undergo oxidation, thereby contributing to ROS neutralization through electron-transfer processes. In substrate-driven catalytic conditions (e.g., H_2_O_2_ presence), the B_1_-Cu nanohybrid system exhibits POD-like activity. This catalytic behavior is consistent with reversible electron-transfer processes involving Cu(I)/Cu(II) redox cycling, as commonly described for copper-based nanozymes. The observed dual function between ROS-scavenging and ROS-generating pathways is expected to depend on the local substrate concentration, surface coverage, and electronic structure of the Cu NCs.

This environment-dependent switch highlights the multifunctional nature of the B_1_-Cu nanohybrid system, where the catalytic function is better described as a dynamic redox balance rather than a single fixed enzymatic function. Based on these findings, the B_1_-Cu nanohybrid system was subsequently evaluated in maize protoplasts to investigate the biological implications of its environment-dependent dual redox behavior.

### 3.3. Effect of B_1_-Cu NCs on the ROS Content, Viability, and Transfection Efficiency of Maize Protoplasts

For the cell treatments, we used so-called maize protoplasts, which are cell wall-removed plant cells. These “naked” cells are crucial in plant biotechnology, as they allow the study of gene introduction (transformation), cell fusion (fusion), and genome editing (e.g., CRISPR). During their isolation, enzymatic degradation of the cell wall leads to the accumulation of reactive oxygen species (ROS) [60], which significantly affects protoplast viability and subsequent callus formation. It has been shown that antioxidant systems can improve protoplast viability and regeneration efficiency [61]. In particular, the application of exogenous antioxidants and redox-active compounds has been widely reported to alleviate oxidative stress during protoplast isolation and culture, thereby enhancing transformation efficiency and regeneration outcomes across different plant systems [62]. For this purpose, we evaluated the Vitamin B_1_ and B_1_-Cu nanohybrid system as potential modulators of intracellular redox homeostasis.

**Figure 5:**
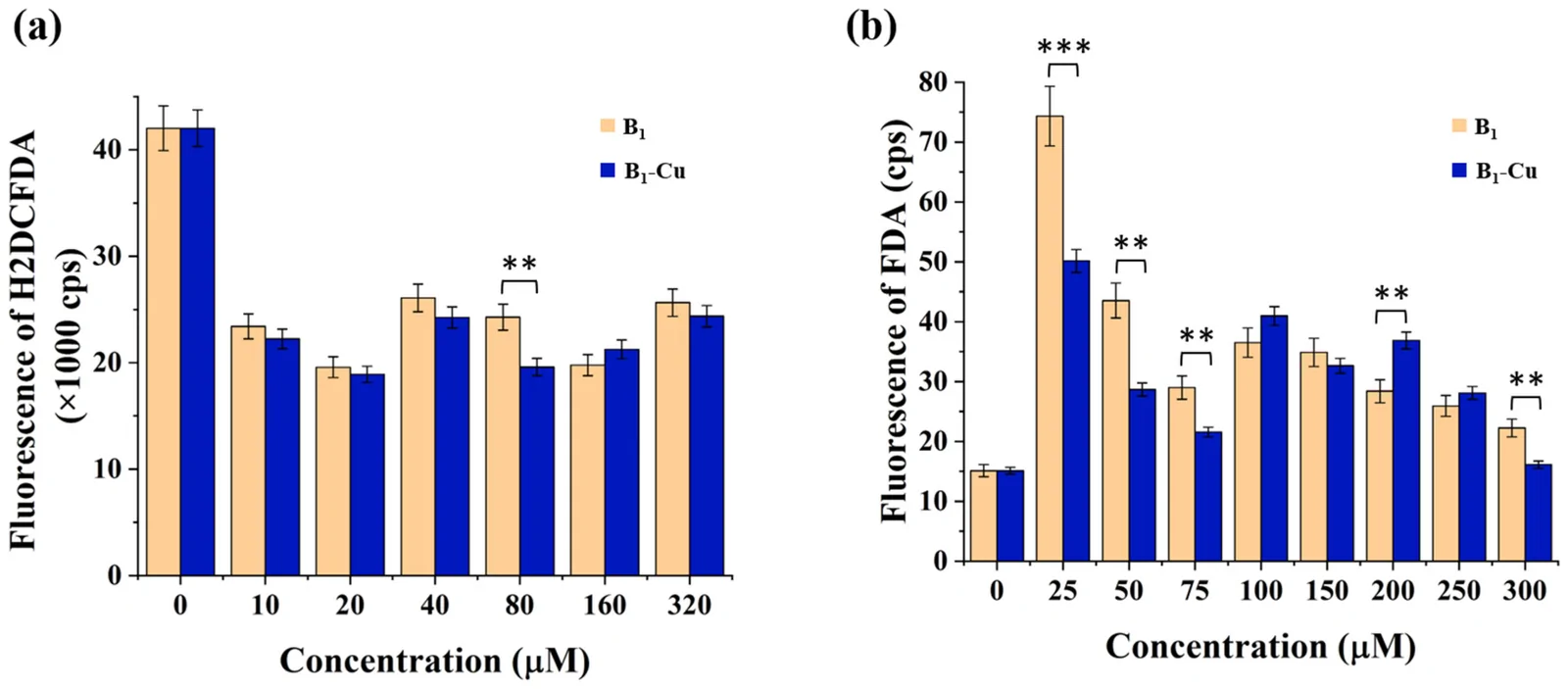
The (**a**) intracellular ROS content and the (**b**) viability of maize protoplasts treated with Vitamin B_1_ or B_1_-Cu nanohybrid system at different concentrations. Data are presented as mean ± SD (*n* = 3 independent biological replicates). Statistical significance between the corresponding Vitamin B_1_ and B_1_-Cu nanohybrid system treatments was determined by using Welch’s *t*-test (** *p* < 0.01, and *** *p* < 0.001). [The applied concentrations refer to the Vitamin B_1_ content in both treatment series].

When the protoplasts were treated with different concentrations of Vitamin B_1_ or the B_1_-Cu nanohybrid system, the ROS content was predominantly decreased compared with the untreated controls (Figure 5a). Vitamin B_1_ exhibited the expected antioxidant effect, where the decrease in intracellular ROS reflects improved redox homeostasis stability, whereas this beneficial effect diminished at higher concentrations. In contrast, the B_1_-Cu nanohybrid system exhibited the characteristics of a redox-active nanozyme capable of modulating intracellular ROS levels. As can be seen in Figure 5a, the measurement data in the case of the B_1_-Cu system display a concentration-dependent response with a distinct activity optimum, indicating an optimal concentration range for redox regulation. At low concentrations, the B_1_-Cu nanohybrid system functions as an effective intracellular ROS buffer. Between 40 and 80 µM Cu NCs concentration, the results indicate a balanced ROS generation-scavenging state (with a statistically significant difference compared with Vitamin B_1_ observed at 80 µM (** *p* < 0.01), whereas higher concentrations were associated with a gradual loss of redox balance. At higher concentrations, the observed response is consistent with saturation of the catalytic process, which may additionally involve ROS-generating reactions associated with copper redox cycling. Importantly, this concentration-dependent ROS regulation is consistent with the dual behavior observed in chemical assays. The B_1_-Cu nanohybrid system exhibits antioxidant activity in ORAC and ABTS measurements, while displaying POD-like activity in the TMB assay. These observations collectively indicate that the system operates as a redox-switchable nanozyme, where apparent antioxidant or pro-oxidant behavior depends on substrate, concentration, and reaction environment, rather than representing distinct mechanistic pathways.

Copper is widely recognized as a double-edged element in plant physiology: it is essential for normal cellular function but becomes toxic when its intracellular concentration exceeds the physiological range [63]. Therefore, the effect of the B_1_-Cu nanohybrid system on maize protoplast viability was investigated. Accordingly, the effects on protoplast viability (Figure 5b) reflect the biological consequences of the concentration-dependent intracellular redox regulation observed after Vitamin B_1_ and B_1_-Cu nanohybrid treatment. At low and intermediate concentrations, both treatments maintained high protoplast viability, suggesting that the applied concentrations were compatible with cellular homeostasis. In contrast, elevated concentrations resulted in decreased viability, indicating a concentration-dependent limitation of cellular tolerance. These effects are consistent with the ROS modulation profile observed by H2DCFDA fluorescence measurements, indicating a close relationship between intracellular ROS regulation and the biological response of the protoplast.

In summary, the prepared B_1_-Cu nanohybrid system exhibits a pronounced hormetic response in maize protoplasts, arising from its environment-dependent redox behavior, which integrates antioxidant and POD-like activities within a single nanozyme platform [64,65].

**Figure 6:**
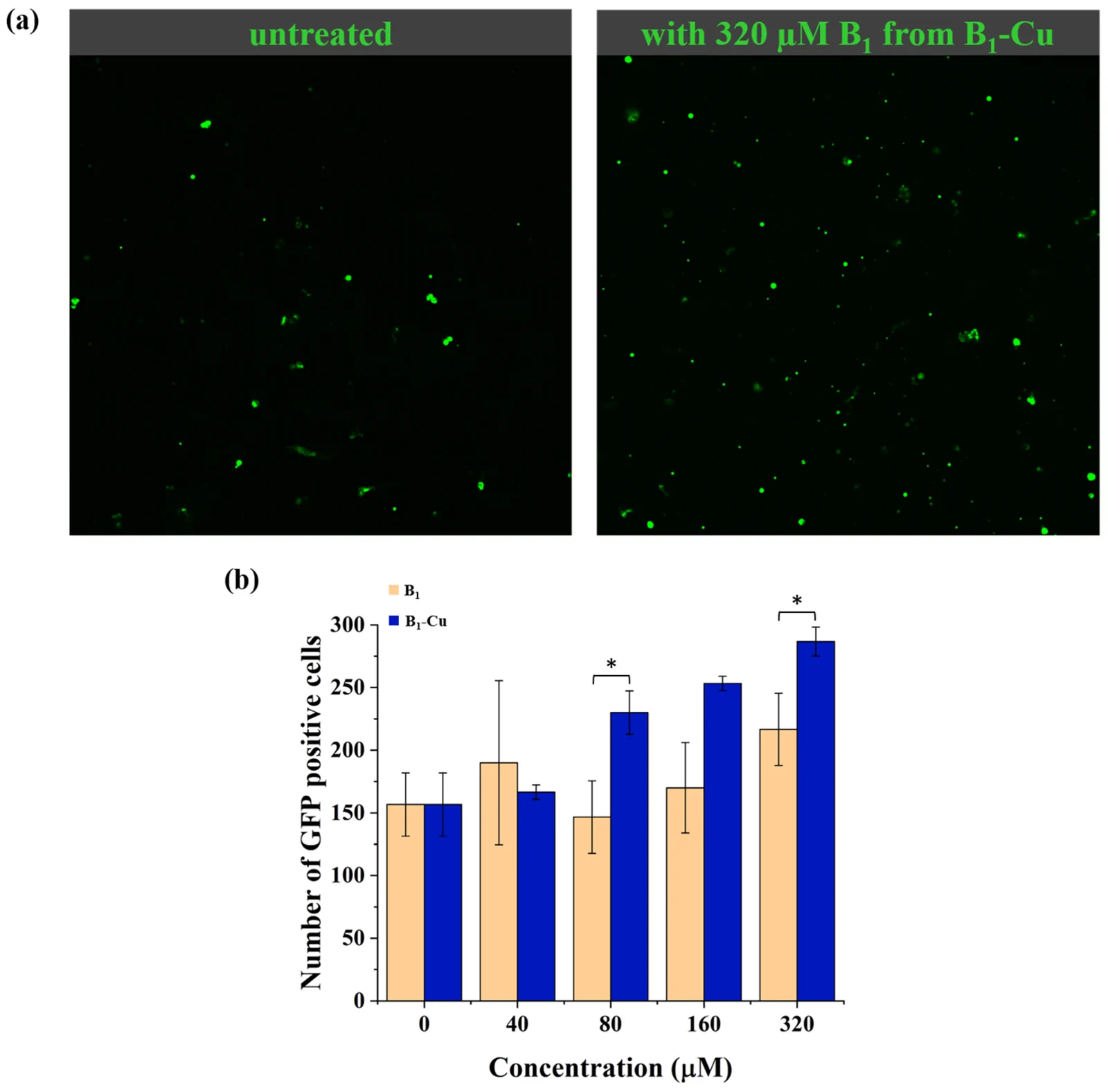
(**a**) Representative fluorescence microscopy images of the untreated (**left**) maize protoplast and protoplast treated with the B_1_-Cu nanohybrid system containing 320 µM Vitamin B_1_ (**right**). (**b**) Number of GFP-positive maize protoplasts following treatment with Vitamin B_1_ or B_1_-Cu NCs at different concentrations. Data are presented as mean ± SD (*n* = 3 independent biological replicates). Statistical significance was evaluated by two-way ANOVA followed by Sidak’s multiple comparisons test as * *p* < 0.05) [The applied concentrations refer to the B_1_ content].

In addition to the antioxidant and viability studies, we investigated the effect of the B_1_-Cu nanohybrid system on maize protoplast transformation efficiency. To facilitate the detection of gene expression, protoplasts were transformed with a plasmid encoding GFP. During the transfection, protoplasts were treated with different concentrations of Vitamin B_1_ or the B_1_-Cu nanohybrid system. The transformed protoplasts were identified by fluorescence microscopy (Figure S5). Figure 6a shows representative fluorescence microscopy images of the protoplasts before and after treatment, where the GFP fluorescence intensity reflects the abundance of viable transformed cells (Figure 6b).

GFP-positive cells were monitored microscopically forming microcalli after 15 days, showing that the treatment did not disrupt cell division. The most important observation from the perspective of maize protoplast applications is that pretreatment with the B_1_-Cu nanohybrid system at concentrations above 80 µM resulted in at least 47% more GFP-positive cells compared to untreated ones. At the highest concentration, the increase in efficacy was even greater: 83% higher than the untreated control and 32% higher than Vitamin B_1_ treatment alone. Therefore, treatment with the B_1_-Cu nanohybrid system significantly increased the number of GFP-positive cells, with statistically significant differences observed at 80 and 320 µM concentrations. This enhancement is consistent with the improved intracellular redox homeostasis established by the dual antioxidant and POD-like activities of the B_1_-Cu nanohybrid system, which may contribute to transient membrane plasticization through dynamic redox regulation [66,67]. Taken together, these findings demonstrate that the B_1_-Cu nanohybrid system not only modulates intracellular redox homeostasis but also translates this effect into a measurable improvement in maize protoplast transformation efficiency.

## 4. Conclusions

In this work, we developed a multifunctional Vitamin B_1_-derived fluorescent Cu nanohybrid system through a simple one-pot synthesis and systematically investigated its structural, redox, and biological properties. The combined spectroscopic, microscopic, and surface analytical characterization demonstrated the formation of ligand-stabilized fluorescent ultrasmall Cu species embedded within a dynamic Cu–thiamine coordination environment. While the precise atomic structure cannot be directly resolved using the applied characterization techniques, the collected experimental evidence consistently supports the formation of fluorescent Cu NCs rather than simple molecular copper complexes.

Beyond the structural characterization, the prepared B_1_-Cu nanohybrid system exhibited a unique environment-dependent dual redox behavior. ORAC and ABTS assays demonstrated a pronounced enhancement of antioxidant activity compared with pure Vitamin B_1_, whereas the TMB assay revealed a distinct pH-dependent peroxidase-like catalytic activity. Together, these results indicate that the same nanohybrid system can function either as an efficient radical scavenger or as a nanozyme depending on the surrounding chemical environment, highlighting its dynamic redox adaptability.

The biological studies further demonstrated that this dual redox behavior translates into intracellular ROS regulation in maize protoplasts. The B_1_-Cu nanohybrid system produced a concentration-dependent hormetic response, maintaining cellular viability within an optimal concentration range while significantly improving transformation efficiency. These observations indicate that controlled modulation of intracellular redox homeostasis represents an effective strategy for enhancing plant cell transformation.

More broadly, this work demonstrates that the functional properties of ligand-stabilized fluorescent copper nanocluster systems are governed not only by the metallic component but also by the chemical nature of the surrounding ligand environment. The present study therefore provides a conceptual framework for the rational design of multifunctional redox-active nanohybrid systems with programmable biological functions. Such systems may find future applications not only in plant biotechnology but also in biosensing, nanozyme engineering, and other biointerface-related fields where precise regulation of intracellular redox processes is required.

## Figures and Tables

**Table 1:** Comparison of the antioxidant activities of representative metal nanoclusters, including the present B_1_-Cu nanohybrid system, determined using different antioxidant assays.

Metal	Stabilization	Test Reaction	Quantitative Value	Ref.
Ag	Bitter ground extract	DPPH	IC_50_ = 12.4 µg mL^−1^	[50]
Au	Vitamin B_1_	ORAC	2.4 ± 0.2 µM TE	[26]
Au	Niacinamide	ORAC	IC_50_ = 18.5 µg mL^−1^	[51]
Pt	Dextran	ORAC	no data	[52]
Au/Ag	Bovine serum albumin	TAC	no data	[11]
Mo	bare particle	ABTS	IC_50_ = 20 µg mL^−1^	[53]
Cu	Chitosan	direct ROS detection	LOD = 15.6 µg mL^−1^	[54]
Cu	Glutathion	DPPH	IC_50_ = 28.2 µg mL^−1^	[21]
Cu	Bis(2-diphenylphosphinophenyl) phosphine	ABTS	dose-dependent	[55]
Cu	Vitamin B_1_	ORAC	229.2 ± 5.8 µM TE	this work
		ABTS	IC_50_ = 14.4 ± 0.4 µM	

## Data Availability

The measurement data are available in the article and the Supplementary Material. The raw data can be requested from the corresponding author.

## Supplementary Materials

The following supporting information can be downloaded at: https://www.mdpi.com/article/10.3390/antiox15081045/s1. Figure S1. (**a**) UV-Vis spectrum of the prepared B_1_-Cu nanohybrid system. (**b**) Comparison of the normalized steady-state excitation and emission spectra of molecular thiochrome (adjusted to pH 3.0 after synthesis) and the B_1_-Cu nanohybrid system measured in aqueous solution under identical pH conditions. The spectra illustrate the distinct optical signatures of the two systems. Figure S2. (**a**) XPS survey spectrum of the B_1_-Cu nanohybrid system. The HR spectra of (**b**) the N 1s, (**c**) O 1 s, (**d**) C 1s, (**e**) S 2p, and (**f**) Cl 2p regions. Scheme S1. Conceptual illustration of a ligand-stabilized fluorescent copper nanocluster model showing a possible coordination environment of oxidized Vitamin B_1_-derived ligands around an ultrasmall copper core. The illustration is intended only to visualize the proposed structure–function relationship inferred from the spectroscopic analyses and does not represent an experimentally resolved atomic structure. Figure S3. (**a**) The TEM image of the dehydrated and aggregated B_1_-Cu nanohybrid system showing aggregated ligand-rich domains containing ultrasmall fluorescent copper species. The effect of pH on (**b**) hydrodynamic diameters (d_H_) and ζ-potential values, and (**c**) the fluorescence emission intensity at 465 nm. (**d**) Representative intensity-weighted DLS size distribution curves of the B_1_-Cu nanohybrid system measured at pH 2.0, 6.0, and 11.0. Table S1. Hydrodynamic diameter (d_H_), the ζ-potential and the polydispersity index (PDI) of the B_1_-Cu nanohybrid system as a function of pH. Figure S4. Maximum absorbance at 450 nm of Vitamin B_1_ (5.4 mM) determined by the stop-bath TMB assay at different pH values. Figure S5. Representative fluorescence microscopy images of the maize protoplasts following treatment with Vitamin B_1_ and the B_1_-Cu nanohybrid system at different concentrations.
