# Supplementary material for: Multifunctional Vitamin B1-Derived Fluorescent Copper Nanoclusters for Efficient Maize Protoplast Transformation
Source: Antioxidants (Basel). 2026 Aug 21;15(8):1045. doi: 10.3390/antiox15081045 (PMC13510130; doi:10.3390/antiox15081045)
Supplement: Supplementary file 1 [file antioxidants-15-01045-s001.zip › antioxidants-4400154-supplementary.pdf]

SUPPORTING INFORMATION

Multifunctional Vitamin B_1-Derived Fluorescent Copper Nanoclusters for Efficient Maize Protoplast Transformation

Nikolett László [1,2], Milán Szabó [1], Györgyi Ferenc [1] and Ditta Ungor [3,*]

1 Institute of Plant Biology, HUN-REN BRC, Temesvári krt. 62, H-6726 Szeged, Hungary
2 Doctoral School of Biology, University of Szeged, Dugonics sqr. 13, H6720 Szeged, Hungary
3 Department of Physical Chemistry and Materials Science, University of Szeged, Rerrich B. sqr. 1, H-6720 Szeged, Hungary; ungord@chem.u-szeged.hu
* Correspondence: ungord@chem.u-szeged.hu

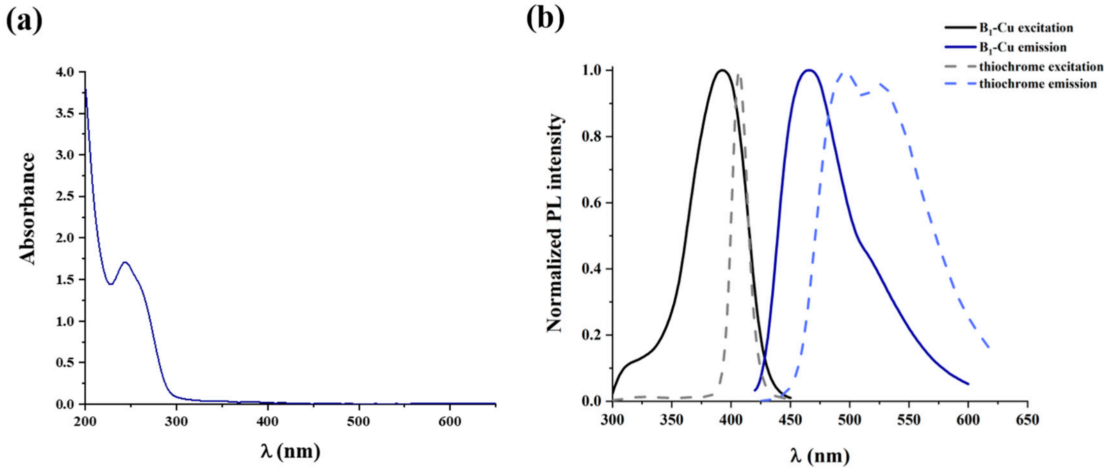

Fig. S1. (a) UV-Vis spectrum of the prepared B_1-Cu nanohybrid system. **(b)** Comparison of the normalized steady-state excitation and emission spectra of molecular thiochrome (adjusted to pH 3.0 after synthesis) and the B_1-Cu nanohybrid system measured in aqueous solution under identical pH conditions. The spectra illustrate the distinct optical signatures of the two systems.

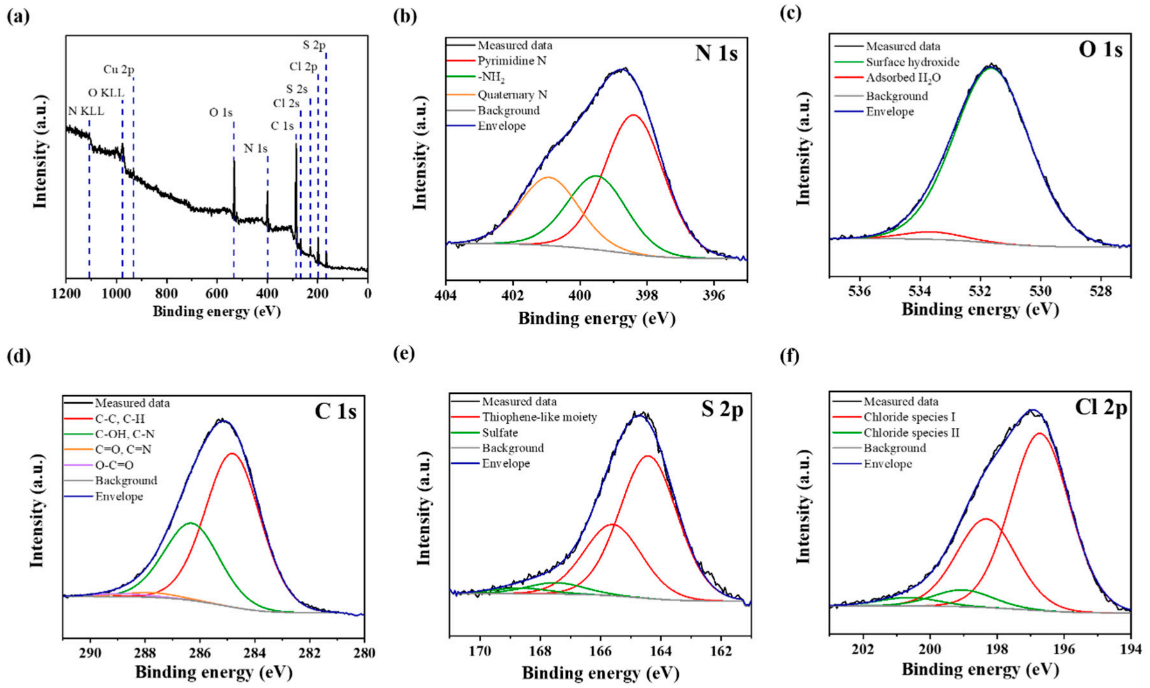

Fig. S2. (a) XPS survey spectrum of the B_1-Cu nanohybrid system. The HR spectra of **(b)** the N 1s, **(c)** O 1 s, **(d)** C 1s, **(e)** S 2p, and **(f)** Cl 2p regions.

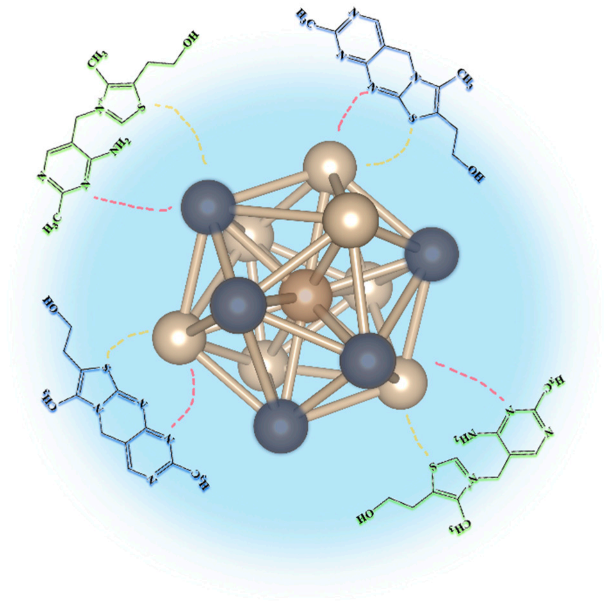

Scheme S1. Conceptual illustration of a ligand-stabilized fluorescent copper nanocluster model showing a possible coordination environment of oxidized Vitamin B_1-derived ligands around an ultrasmall copper core. The illustration is intended only to visualize the proposed structure–function relationship inferred from the spectroscopic analyses and does not represent an experimentally resolved atomic structure.

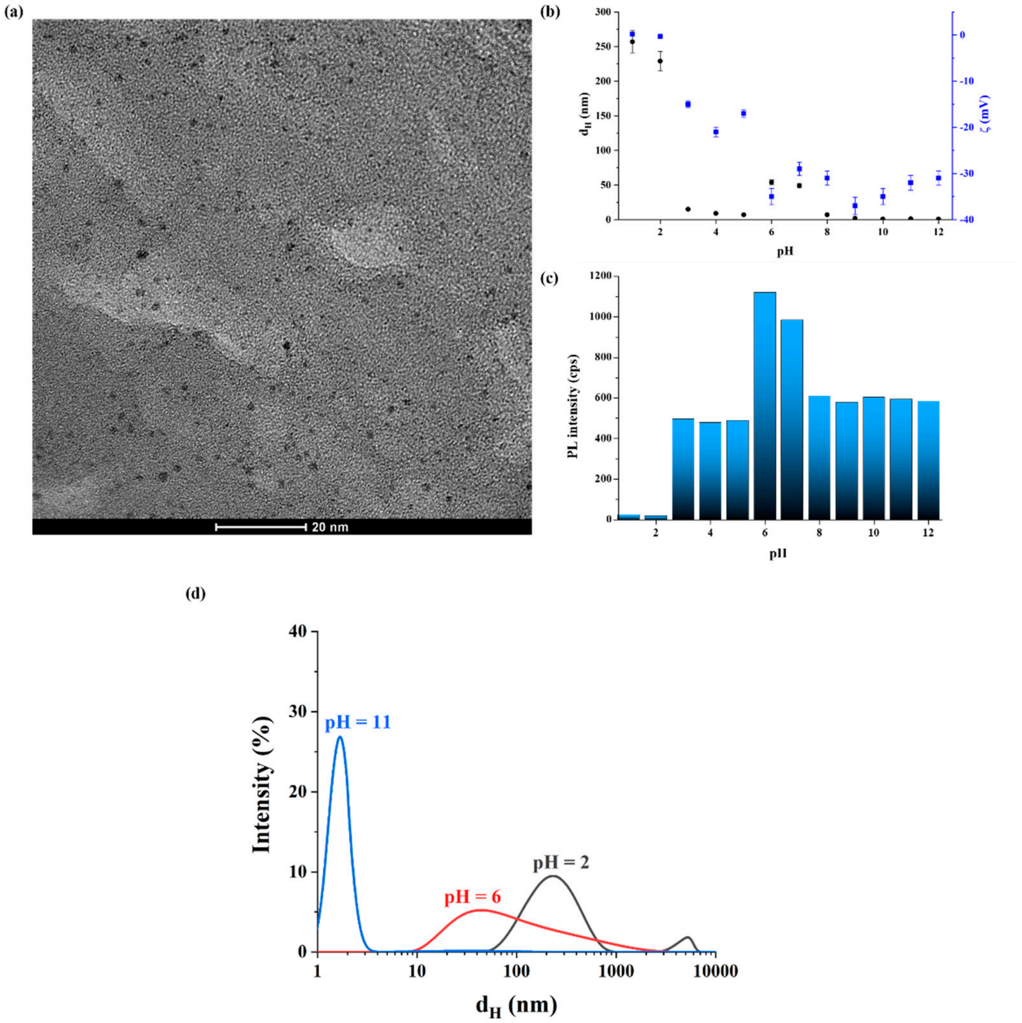

Fig. S3. (a) The TEM image of the dehydrated and aggregated B_1-Cu nanohybrid system showing aggregated ligand-rich domains containing ultrasmall fluorescent copper species. The effect of pH on **(b)** hydrodynamic diameters (d_H) and ζ-potential values, and **(c)** the fluorescence emission intensity at 465 nm. **(d)** Representative intensity-weighted DLS size distribution curves of the B_1-Cu nanohybrid system measured at pH 2.0, 6.0, and 11.0.

Table S1. Hydrodynamic diameter (d_H), the ζ-potential and the polydispersity index (*PDI*) of the B_1-Cu nanohybrid system as a function of pH.

pH	dH (nm)	PDI	ζ-potential (mV)
1	257.2 ± 15.9	0.512	0.2 ± 0.0
2	229.3 ± 14.2	0.504	-0.3 ± 0.1
3	15.0 ± 0.9	0.351	-15.1 ± 0.75
4	9.0 ±0.5	0.204	-21.2 ± 1.01
5	7.0 ±0.4	0.201	-17.01 ± 0.85
6	54.1 ± 3.4	0.221	-35.0 ± 1.7
7	49.2 ± 3.1	0.362	-29.2 ± 1.5
8	7.1 ± 0.4	0.298	-31.03 ± 1.5
9	2.0 ± 0.1	0.200	-37.0 ± 1.8
10	0.9 ± 0.1	0.362	-35.1 ± 1.5
11	1.3 ± 0.1	0.301	-32.3 ± 1.7
12	0.8 ± 0.1	0.215	-31.0 1.5

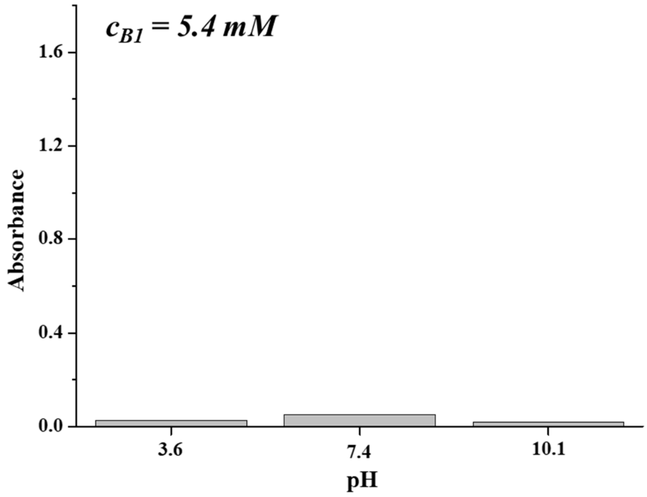

Fig. S4. Maximum absorbance at 450 nm of Vitamin B_1 (5.4 mM) determined by the stop-bath TMB assay at different pH values.

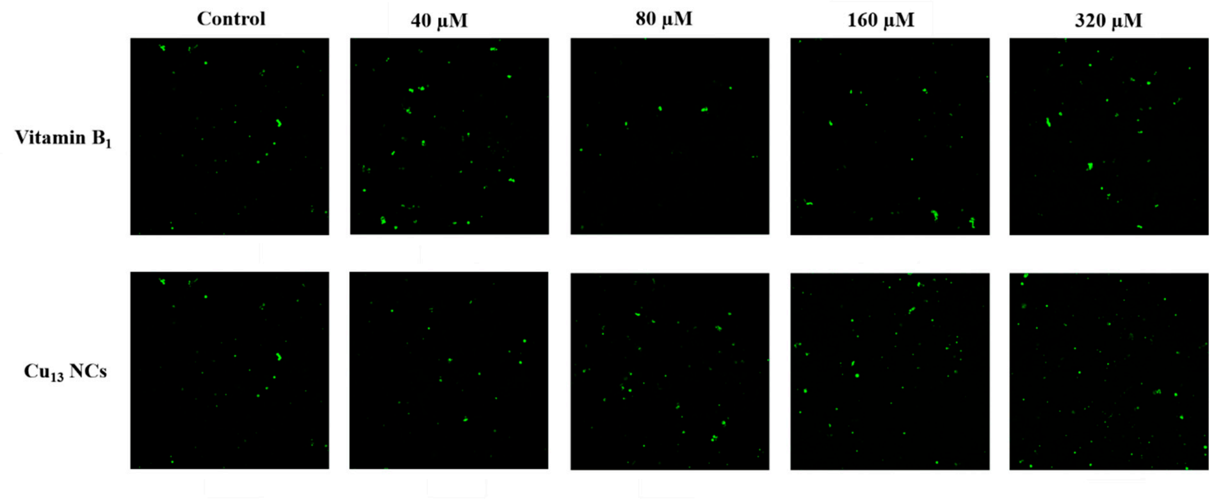

Fig. S5. Representative fluorescence microscopy images of the maize protoplasts following treatment with Vitamin B_1 and the B_1-Cu nanohybrid system at different concentrations.
